# Impaired Ethanol-Induced Sensitization and Decreased Cannabinoid Receptor-1 in a Model of Posttraumatic Stress Disorder

**DOI:** 10.1371/journal.pone.0155759

**Published:** 2016-05-17

**Authors:** Jessica J. Matchynski-Franks, Laura L. Susick, Brandy L. Schneider, Shane A. Perrine, Alana C. Conti

**Affiliations:** 1 Research Service, John D. Dingell VA Medical Center, Detroit, Michigan, United States of America; 2 Department of Neurosurgery, Wayne State University School of Medicine, Detroit, Michigan, United States of America; 3 Department of Psychiatry and Behavioral Neurosciences, Wayne State University School of Medicine, Detroit, Michigan, United States of America; University of Florida, UNITED STATES

## Abstract

**Background and Purpose:**

Impaired striatal neuroplasticity may underlie increased alcoholism documented in those with posttraumatic stress disorder (PTSD). Cannabinoid receptor-1 (CB1) is sensitive to the effects of ethanol (EtOH) and traumatic stress, and is a critical regulator of striatal plasticity. To investigate CB1 involvement in the PTSD-alcohol interaction, this study measured the effects of traumatic stress using a model of PTSD, mouse single-prolonged stress (mSPS), on EtOH-induced locomotor sensitization and striatal CB1 levels.

**Methods:**

Mice were exposed to mSPS, which includes: 2-h restraint, 10-min group forced swim, 15-min exposure to rat bedding odor, and diethyl ether exposure until unconsciousness or control conditions. Seven days following mSPS exposure, the locomotor sensitizing effects of EtOH were assessed. CB1, post-synaptic density-95 (PSD95), and dopamine-2 receptor (D2) protein levels were then quantified in the dorsal striatum using standard immunoblotting techniques.

**Results:**

Mice exposed to mSPS-EtOH demonstrated impaired EtOH-induced locomotor sensitization compared to Control-EtOH mice, which was accompanied by reduced striatal CB1 levels. EtOH increased striatal PSD95 in control and mSPS-exposed mice. Additionally, mSPS-Saline exposure increased striatal PSD95 and decreased D2 protein expression, with mSPS-EtOH exposure alleviating these changes.

**Conclusions:**

These data indicate that the mSPS model of PTSD blunts the behavioral sensitizing effects of EtOH, a response that suggests impaired striatal neuroplasticity. Additionally, this study demonstrates that mice exposed to mSPS and repeated EtOH exposure decreases CB1 in the striatum, providing a mechanism of interest for understanding the effects of EtOH following severe, multimodal stress exposure.

## Introduction

A high comorbidity exists between posttraumatic stress disorder (PTSD) and alcoholism [1]. Specifically, among military personnel, over 60% of those that develop an alcohol use disorder during or after deployment are also diagnosed with PTSD [2]. Like other addictions, the severity of alcoholism is exacerbated by stress [3–6], however the mechanisms of this underlying interaction are not fully understood. Those with PTSD and alcoholics display abnormal striatal activity [7–12], suggesting dysregulated striatal neuroplasticity may underlie the effects of PTSD on alcohol-associated addictive behaviors.

The endocannabinoid system, including cannabinoid receptor-1 (CB1) and its endogenous endocannabinoid ligands, anandamide and 2-arachidonoylglycerol (2-AG), is an important modulator of dorsal striatal neuroplasticity and has known involvement in fear processing and PTSD diagnoses. Endocannabinoids regulate long-term depression (LTD) of pre-synaptic neurons through retrograde activation of CB1 receptors [13], which are found on a variety of cell types throughout the brain including both gamma-aminobutyric acid (GABA) interneurons and glutamatergic terminals in the dorsal striatum [14]. The endocannabinoid system is particularly important in regulating fear extinction [15, 16], modulating the hypothalamic-pituitary-adrenal axis [17–20], and influencing amygdala habituation to fear as well as stress-reactivity personality traits [21]. Cannabis use amongst those with PTSD is elevated, which is at least partly motivated by symptom coping [22–24]. Neumeister and colleagues identified elevated CB1 availability throughout the brain, including the caudate, and decreased peripheral anandamide levels in PTSD patients suggesting altered endocannabinoid tone [25]. Additionally, a small clinical trial documented that a CB1 agonist improved nightmare-related symptoms and reported well-being of those with PTSD [26]. Those with PTSD exhibit dysregulation of the endocannabinoid system [25, 27], similar to that observed in rodent stress models, including the rat single-prolonged stress (SPS) model of PTSD (for review see [17]). The SPS rat, and the modified mouse SPS (mSPS) models of PTSD both cause neuroendocrine changes that lead to enhanced negative feedback of the hypothalamic-pituitary-adrenal axis, and impaired fear extinction [28, 29]. These behavioral and endocrine changes that reflect symptoms of PTSD, were prevented by injection of a CB1 receptor agonist peripherally or directly into the amygdala [30] or hippocampus [31] within 24 h of SPS exposure. Similarly, genetic or drug-induced enhancement of endocannabinoids reduced behavioral symptoms, endocrine responses, and dendritic hypertrophy in a variety of PTSD-like models [17, 30, 31].

CB1 receptors are also involved in regulating EtOH response, are impacted by EtOH, and those within the dorsal striatum appear to especially affect EtOH preference in pre-clinical studies. In an electrophysiology study, CB1 receptor agonists attenuated the effects of EtOH on GABAergic activation of the central amygdala [32]. In addition, activation of CB1 receptors is required for the EtOH-induced depression of glutamatergic neurons in both the dorsal striatum and the hippocampus [13, 33–35]. Clinical studies indicate a decrease in CB1 and dysregulation of endocannabinoids in the ventral striatum of alcoholics [36]. Similarly, preclinical studies indicate decreases in CB1 receptor levels, G-protein binding to CB1 receptors, and increases in 2-AG within the dorsal striatum after EtOH vapor exposure in mice [37, 38], as well as a decrease in CB1 signaling after chronic intermittent voluntary EtOH drinking in rats [39]. Interestingly, while CB1 receptor antagonists have been shown to increase EtOH preference if given during a 30-day EtOH exposure, they decrease EtOH preference if given after 30 days of EtOH exposure [40].

In conjunction with CB1 receptor dysregulation, chronic intermittent EtOH vapor exposure leads to dendritic hypertrophy within the mouse striatum, as well as impaired striatal learning in mice [37]. In mouse studies, psychomotor sensitization to EtOH [41, 42] may be representative of neuroplastic change [43], is related to stress-associated mechanisms [43], and is considered to be striatal-dependent [44]. In this procedure, mice are exposed to repeated administration of a locomotor activity (LMA)-inducing dose of EtOH, and over time, display an increase in behavioral sensitivity to the drug [41, 42]. CB1 receptor involvement in EtOH locomotor sensitization is not fully understood, however Coelhoso and colleagues have shown that high-responders to EtOH-sensitization show increased CB1 receptors [45] and numerous reports suggest endocannabinoids, their receptors, and catabolic enzyme, fatty acid amide hydrolase (FAAH), are critical in the response to EtOH and EtOH preference [40, 45–49]. This CB1-EtOH interaction is likely due to CB1 receptor regulation of LTD on both GABAergic and glutamatergic targets within the striatum (for review [50]).

Additionally, there is an established modulatory relationship between striatal dopamine-2 (D2) and CB1 receptors. The *in vivo* knockdown of either receptor in the striatum, leads to decreased protein expression, mRNA expression, and receptor-mediated G-protein activation of the opposing receptor [51]. This modulatory relationship also appears to impact acute striatal signaling [52, 53]. In D2 receptor expressing medium spiny neurons (MSNs), activation of D2 receptors increases the release of endogenous anandimide, while CB1 receptor antagonists increases spontaneous excitatory postsynaptic currents (sEPSC) and partially prevents D2-agonist induced decreases in EPSCs in these MSNs [52, 53]. Additionally, CB1 receptors are required for pre-synaptic striatal D2 autoreceptor inhibition of glutamatergic neurons [54]. D2 receptors are also involved in the response to EtOH, however their involvement appears to be complex and is not fully understood. Deletion of D2 receptors decreases EtOH preference [55] and the antagonism of striatal D2 receptors *in vivo* decreases habit-based EtOH-seeking in mice [56]. In contrast, overexpression of D2-receptors in the ventral striatum reduces EtOH preference in rats [57], D2 receptor agonists decrease EtOH preference of mice [58], and striatal D2-receptor binding positively correlates with behavioral sensitization to EtOH in mice [44]. In clinical studies, alcoholics show decreases in D2 receptors, however there is some variance in these reports [59, 60]. D2 receptors are also related to traumatic stress exposure. Enman and colleagues found decreased D2 protein in the striatum and nucleus accumbens of rats exposed to SPS [61]. Genes related to D2 receptors have also been associated with increased risk of PTSD, but the results are varied (for review see [62]). Interestingly, this D2-related genetic risk factor appears to be specifically relevant to cases that also include heavy alcohol consumption, indicating a possible mechanism of the PTSD-alcoholism association [63].

Post-synaptic density 95 (PSD95) has also been implicated in the effects of EtOH and stress exposure, and has recently been associated with the endocannabinoid system. As a post-synaptic scaffolding protein, PSD95 is important in regulating neuroplastic changes through its ability to accumulate N-methyl D-aspartate (NMDA) receptors on post-synaptic neurons [64, 65]. It is also important in balancing homeostatic disruptions in neuronal excitation and inhibition, specifically observed in drug responses [64, 65]. An *in vitro* study of PSD95 type 3 in MSNs, the most common type of PSD95 within the striatum [66], indicates that PSD95 type 3 is important in regulating endocannabinoid striatal plasticity through its modulation of glutamatergic receptor-5 (mGlu5) [67]. When striatal MSN mGlu5 is activated, there is a post-synaptic release of endocannabinoids, activating presynaptic CB1 receptors and inducing LTD of pre-synaptic glutamatergic cortical-striatal neurons [50]. Studies show that PSD95-related genes are upregulated in the lower midbrain of mice in response to *in vivo* EtOH vapor exposure [68], is important in fear conditioning [69] and is increased in several stress models [70, 71]. However, the effects of EtOH and/or severe multimodal stress on regulating striatal PSD95 and endocannabinoid interactions remain unidentified.

In order to define more clearly the striatal mechanisms involved in the PTSD-alcohol interaction, the present study measured the effects of severe, multimodal stress exposure using the mSPS model of PTSD on EtOH-induced locomotor sensitization, as well as on striatal CB1, D2, and PSD95 protein levels. It was hypothesized in the present study that mSPS would increase EtOH-induced locomotor sensitization in association with a decrease in CB1 and D2 and an increase in PSD95 protein expression in the dorsal striatum.

## Materials and Methods

### Mice

Male C57BL/6 mice (*mus musculus*) were housed in groups of 2–5 mice with standard mouse chow and water available *ad libitum* when not being tested. All procedures were approved by the Wayne State University Institutional Animal Care and Use Committee. Mice were tested in 2 cohorts, where the first (BEC-Test) were treated in the test room and trunk blood was utilized for blood ethanol content (BEC) (*n* = 36), divided equally into 4 groups, naïve mice treated with saline (Control-Sal), naïve mice treated with EtOH (Control-EtOH), mSPS-exposed mice treated with saline (mSPS-Sal), and mSPS-exposed mice treated with EtOH (mSPS-EtOH). The second cohort was treated in their home cages and their brains were utilized for immunoassays (Immuno-Home) (*n* = 42), with groups including Control-Sal (*n* = 10), Control-EtOH (*n* = 11), mSPS-Sal (*n* = 10), and mSPS-EtOH (*n* = 11).

### Mouse Single Prolonged Stress (mSPS)

**Fig 1:**
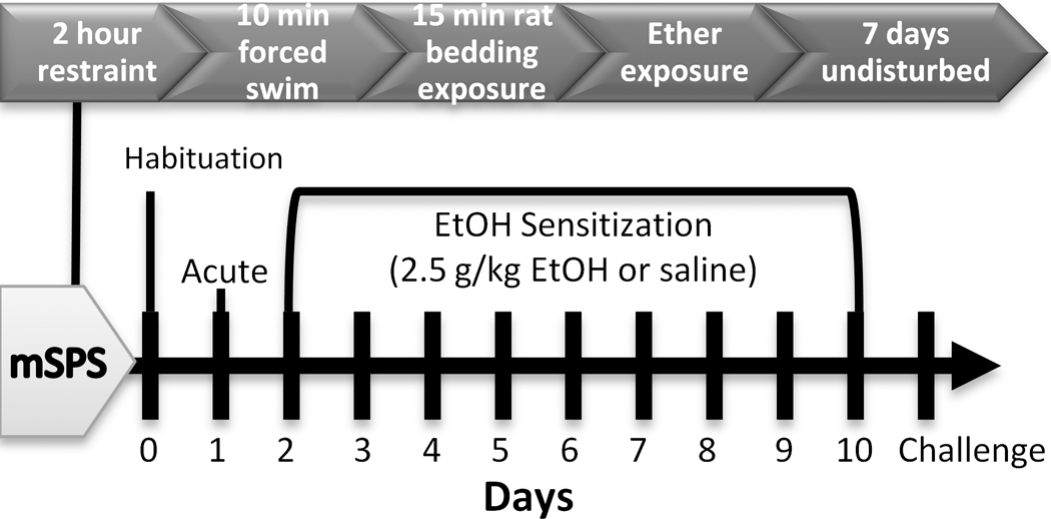
The above timeline of events summarizes the mSPS and EtOH sensitization paradigms.

mSPS procedures were adapted from Eagle and colleagues [28] and outlined with all other behavioral procedures in Fig 1. Briefly, mSPS mice were habituated to the testing room for 1 h, and then were individually restrained by loose immobilization in ventilated 50 mL tubes for 2 h. Next, the mSPS mice were exposed to a forced group swim for 10 min, where 4–5 randomly selected mice swam in cylinders (18 cm diameter) filled with water (25°C) to 14 cm. After the forced group swim, the mice were quickly towel dried and placed into home cages. Next, during a 15 min rest period, the mice were exposed to odors from soiled rat bedding as a predator scent. Lastly, the mSPS mice were exposed to diethyl ether on soaked cotton balls (no direct contact) in an enclosed chamber until loss of consciousness (approximately 5 min), and placed on warming pads until active and alert. Control mice were brought to a separate testing room for 2 h where they were weighed and handled. All mice were then returned to the vivarium and left undisturbed for a total of 7 days after mSPS or control exposure, aside from 1 cage-change on day 5 post-exposure.

### Ethanol Sensitization

In previous sensitization studies in our laboratory, mice received injections in the test room on habituation, acute-EtOH and challenge days, while injections in between the acute and challenge days were conducted in their home cages. In order to confirm that sensitization was not confounded by a change in context, we tested the mice in 2 cohorts. First, in the BEC-Test cohort the mice were always injected in the testing room and received daily exposure to the LMA chambers on days 2–10. After the completion of the BEC-Test cohort’s assessments, the Immuno-Home cohort began testing. The Immuno-Home cohort was injected in the test room on days when LMA was assessed (habituation, acute-EtOH, and challenge), but was injected in the home cages on days 2–10 as done in previous work.

On day 8 post mSPS or control condition exposure, all mice were habituated to the OPTO-M3 LMA chambers (Coulborn Instruments, Whitehall, PA). The LMA chambers have 16 light beam sensors on the x-axis, with 2.46 cm between each sensor, which scan at a rate of 160 Hz in the arena measuring 21.5 x 44 cm. The LMA chambers were kept in isolation cubicles with a house fan for ventilation (62 db) and overhead lighting (approximately 200 lumens). Mice were injected intraperitoneally (i.p. 0.01 mL/g body weight) with sterile saline (0.9% NaCl) then immediately placed into the LMA chambers where total activity (total beam breaks) was measured. The first 50 minutes served as habituation to the apparatus and the final 10 minutes served as their baseline activity. Mice were then returned to their home cages. The following day the mice began Day 1 of EtOH sensitization. On Day 1, the mice were injected with 2.0 g/kg EtOH (20% v/v, i.p.) dissolved in saline or saline alone as a control. LMA was immediately assessed for 10 min. On Days 2–10 of EtOH sensitization, the same procedure was repeated, except mice receiving EtOH were administered a 2.5 g/kg dose. Finally, on Day 11 of EtOH sensitization, EtOH groups were injected with a challenge dose of 2.0 g/kg of EtOH and all animals were tested for LMA for 10 min.

### Sample Extraction & Preparation

Immediately following the final LMA test session, the mice were euthanized by cervical dislocation, and brains were removed and stored at -80°C until microdissection to obtain striatal tissue samples. Trunk blood samples from the BEC-Test cohort were also collected and centrifuged at 5,000 rpm for 10 min to obtain plasma, which was stored at -80°C until use. Brains from the Immuno-Home cohort were sliced into 2 mm sections using a rodent brain slicer matrix (Zivic Instruments, Pittsburgh, PA), and unilateral 1.5 mm tissue punches (Miltex, Inc., York, PA) from the left anterior-dorsal striatum at 1.5 to -0.5 mm anterior to bregma were taken and stored at -80°C until immunoassay. A unilateral punch was then homogenized for approximately 20 seconds in lysis buffer (20 mM Tris pH 7.5, 150 mM NaCl, 2mM EDTA, 1% triton X-100, 10% glycerol with Phosphatase Inhibitor Cocktail 2 P5726, Phosphatase Inhibitor Cocktail 3 P0044 and Protease Inhibitor P8340 from Sigma Aldrich, St. Louis, MO), then centrifuged at 4°C for 10 min at 10,000 x g, and the protein was quantified using a Pierce 660 nm protein assay using a bovine serum albumin standard (Thermo Scientific, Rockford, IL).

### Blood Ethanol Content

Samples from the BEC-Test cohort were diluted 1:1 with saline. Solutions containing 0.5% EtOH standard, saline control, or sample in Alcohol Reagent Set according to manufacturer instructions (Pointe Scientific Inc., Canton, MI) were incubated at 37°C for 5 min, and then measured at 340 nm using a BioMate 3S UV-Visible Photospectrometer (Thermo Scientific, Rockford, IL). One mSPS-EtOH sample was not included due to insufficient plasma collection.

### Immunoblotting

Immunoblotting was conducted as described previously [72]. Protein (20 μg) was separated on a NuPAGE 4–12% Bis-Tris Gel using MOPS running buffer, and then transferred to a nitrocellulose membrane using NuPAGE transfer buffer on a semi-dry transfer apparatus at 15 V for 40 min (all from Life Technologies, Carlsbad, CA). One membrane was prepared for detection of CB1, PSD95 and actin, with a second membrane prepared for detection of D2 and actin proteins. After 3x10 min rinses in Tris-buffered saline containing 0.1% Tween 20 (TBST), nonspecific binding was blocked for 1 h using 5% non-fat dry milk in TBST. Antibodies against CB1 (1:1000; 10006590; Cayman Chemical, Saxtons River, VA), D2 (1:2000; Alomone labs, Jerusalem, Israel), PSD95 (1:5000; MA1-045; Thermo Scientific, Waltham, MA), and actin (1:5000; A5060, Sigma-Aldrich, St. Louis, MO) were applied individually overnight in a solution of TBST and 5% dry milk. CB1, D2, and actin were detected using 1:1000, 1:2000, or 1:2000 anti-rabbit IgG HRP-linked antibody, respectively (7074, Cell Signaling Technology, Beverly, MA). PSD95 was detected using 1:5000 anti-mouse IgG HRP-linked antibody (NA931V; GE Healthcare UK Limited, Little Chalfont Buckinghamshire HP7 9NA, UK). All secondary antibodies were developed with Super Signal West Dura Extended Duration Substrate (Thermo Scientific, Waltham, MA). Membranes were stripped of proteins between exposures to antibodies using Restore TM PLUS Western Blot Stripping Buffer (Thermo Scientific, Waltham, MA) for 2 min followed by 3 x 1 sec rinses followed by 3 x 10 min rinses in TBST according to manufacturer instructions. Densiometric analysis using Image J software (NIH) was used to quantify CB1, D2, and PSD95 immunoblot signals which were normalized to actin.

### Statistical Analyses

All analyses were conducted using IBM Statistical Package for the Social Sciences (SPSS) version 22 (IBM Corporation, Armonk, NY). Three-way mixed-design analyses of variance (ANOVA) were used to determine if within-subjects factor LMA sensitization test day (day 1, challenge) and between subjects factors EtOH (Saline, EtOH) and mSPS (Control, mSPS) had effects on the dependent variables, raw LMA and percent baseline LMA. An independent-t test was used to determine if mSPS had an effect on BECs. Two-way ANOVAs were used to assess the effects of EtOH and mSPS on striatal CB1, PSD95, and D2. Pearson’s partial correlation coefficient analyses were utilized to determine relationships between percent baseline LMA on challenge and striatal CB1. Alpha was set at 0.05 for all analyses.

## Results

### EtOH-induced LMA Sensitization and Blood Ethanol Content

Because the cohort*day interaction was not significant in the BEC-Test and Immuno-Home control groups, the cohorts were analyzed as a single study, *F*(1,70) = 0.350, *p* = 0.556. Two mSPS-exposed mice died, 1 during forced-swim and 1 after ether exposure. Additionally, Control-Sal (n = 1), Control-EtOH (n = 2), and mSPS-EtOH (n = 2) were removed due to a technical malfunction during data collection in LMA. Final groups included: Control-Sal (*n* = 18), Control-EtOH (*n* = 18), mSPS-Sal (*n* = 18), and mSPS-EtOH (*n* = 18), for a total (*n* = 72).

**Fig 2:**
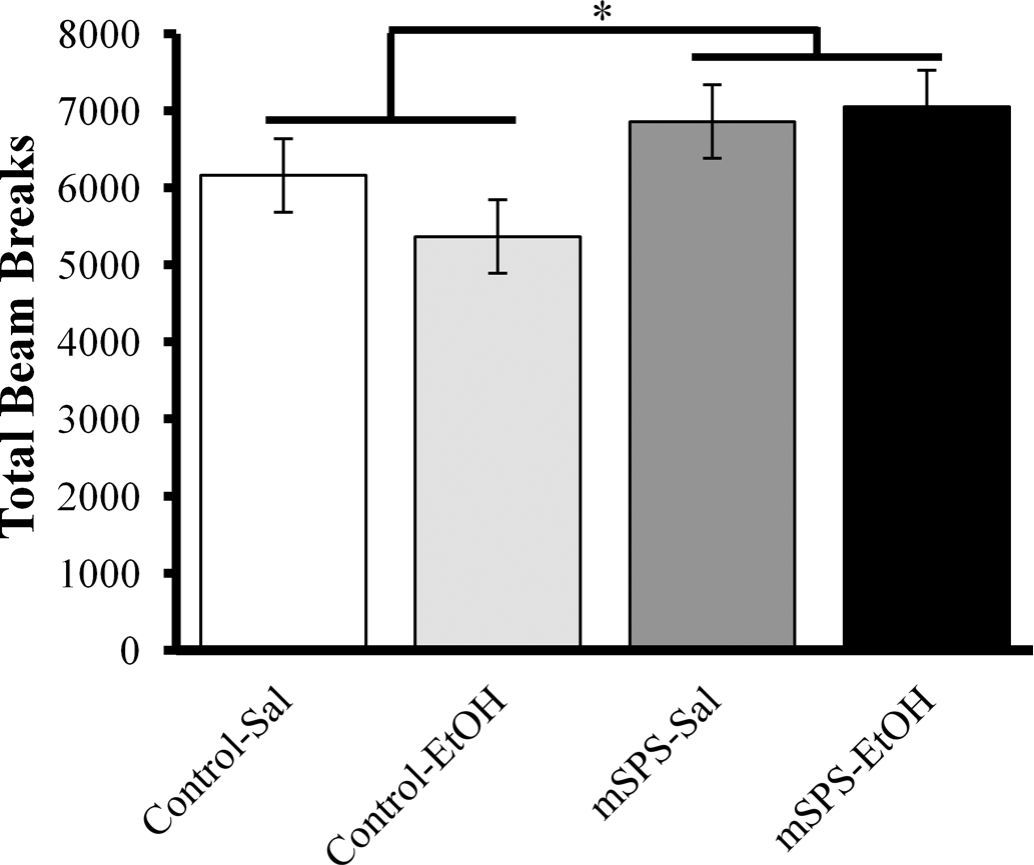
Activity was significantly increased during habituation in mSPS-exposed mice compared to control mice prior to EtOH exposure (*p<0.05).

A two-way ANOVA revealed an overall effect of mSPS during habituation, which indicated that mSPS-exposed mice were significantly more active compared to control mice, *F*(1,68) = 6.286, *p* = 0.015 (Fig 2). Because the mSPS and control groups were significantly different prior to EtOH exposure, and to provide a more representative visualization, the last 10 minutes of the LMA habituation were used to calculate percent baseline LMA when assessing the EtOH sensitization paradigm in addition to assessments of raw data.

**Fig 3:**
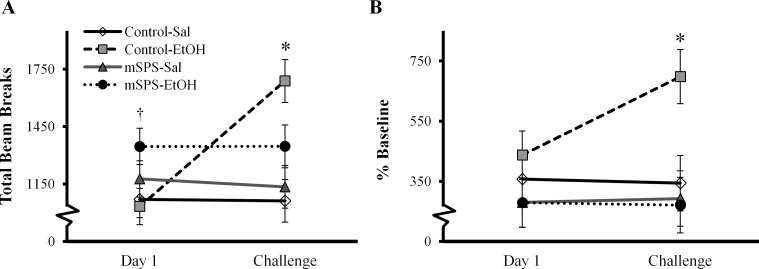
Mice exposed to mSPS followed by chronic EtOH (mSPS-EtOH) demonstrate impaired EtOH-LMA sensitization. *p<0.05 Control-EtOH mice on challenge compared to Control-Sal on Day 1, as well as Control-Sal and mSPS-EtOH on Challenge. ^†^*p*<0.05 mSPS-EtOH mice compared to Control-EtOH mice on Day 1.

**Fig 4:**
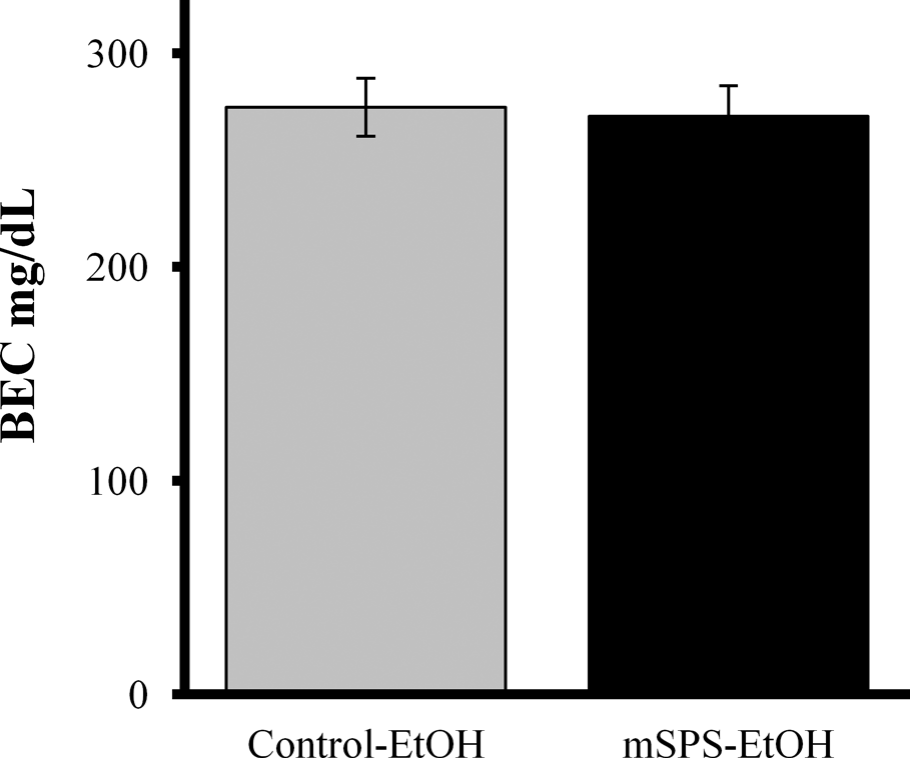
mSPS did not significantly alter BEC compared to control (*p*>0.05).

When assessing raw LMA, a three-way ANOVA also revealed a significant mSPS*EtOH*Day interaction, *F*(1, 68) = 5.943, *p* = 0.017, with increases in Control-EtOH mice on challenge compared to Control-EtOH mice on Day 1, *p*<0.001, Control-Sal mice on challenge, *p*<0.001, and mSPS-EtOH on challenge, *p* = 0.034, as well as a significant increase in mSPS-EtOH mice compared to Control-EtOH mice on Day 1, *p* = 0.034 (Fig 3). A three-way ANOVA also revealed a significant mSPS*EtOH*Day interaction in percent baseline LMA assessed in the sensitization paradigm, *F*(1, 68) = 12.664, *p* = 0.001 (Fig 3). Percent baseline LMA activity was significantly increased in Control-EtOH mice on challenge compared to Control-EtOH mice on Day 1, *p*<0.001, Control-Sal mice on challenge, *p* = 0.011, and mSPS-EtOH on challenge, *p* = 0.002. An independent t-test indicated that BEC did not differ significantly between mSPS-EtOH and Control-EtOH mice in the BEC-Test cohort, *t*(16), *p* = 0.749 (Fig 4).

### Immunoblot Assays

**Fig 5:**
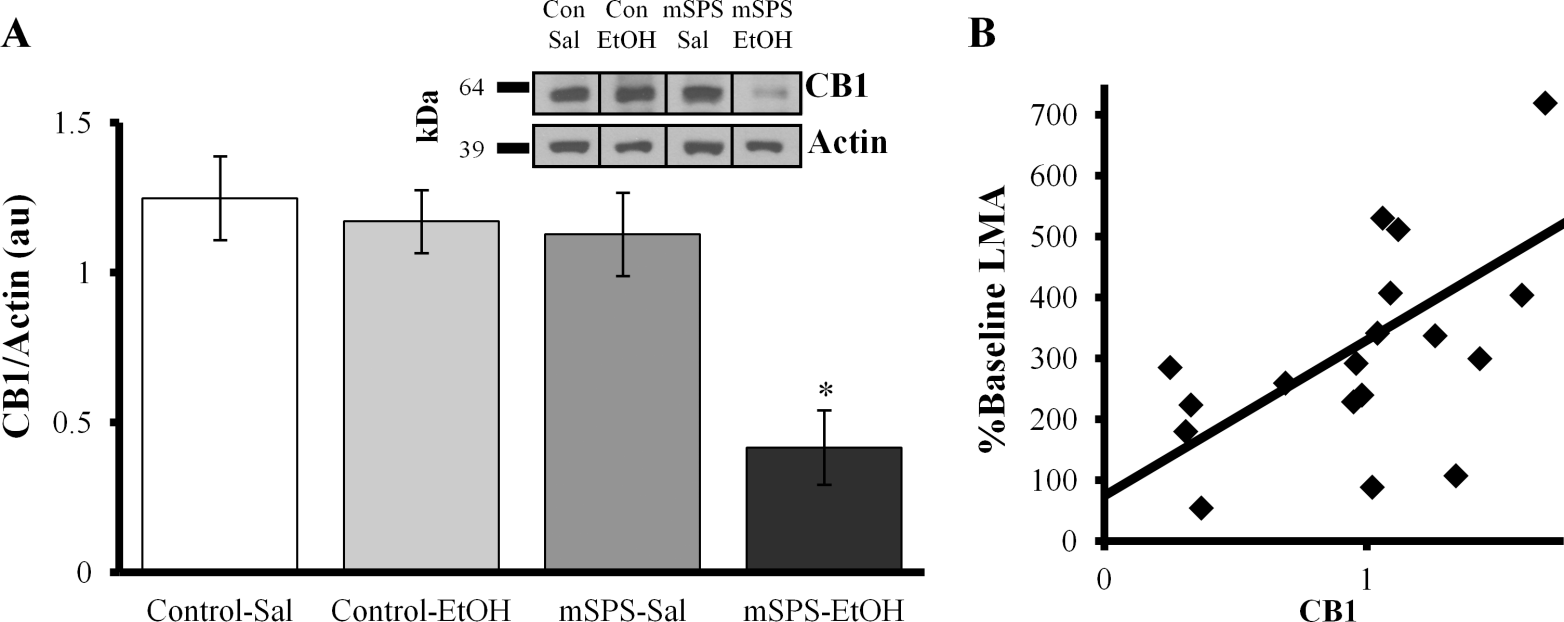
CB1 receptor protein was significantly decreased in mice exposed to mSPS followed by chronic EtOH in the anterior striatum compared to all other treatments. (A) *p<0.05; au, arbitrary units. Representative samples inserted in upper right corner (kDa, kilodaltons). (B) LMA movements at challenge compared to LMA during last 10 min of habituation (% Baseline) positively correlated with striatal CB1 receptor protein (*p*<0.05), suggesting that LMA decreased as CB1 receptors decreased.

A total of 20 samples from the Immuno-Home cohort were utilized for CB1 assays (2 samples (1 Control-Sal and 1 mSPS-EtOH) were removed from the original 22 samples due to insufficient protein collection). Final groups included, Control-Sal (*n* = 4), Control-EtOH (*n* = 7), mSPS-Sal (*n* = 4), and mSPS-EtOH (*n* = 5). Analysis of CB1 protein levels with a two-way ANOVA revealed a significant mSPS*EtOH interaction, *F*(3,16) = 6.169, *p* = 0.024 (Fig 5A). Pair-wise comparisons indicated that mSPS-EtOH mice showed significantly decreased CB1 compared to Control-EtOH mice, *p*<0.001, and to mSPS-Sal mice, *p* = 0.002. CB1 positively correlated with percent baseline LMA, such that percent baseline LMA decreases as CB1 receptor levels decrease, *r*(18) = 0.581, *p* = 0.009 (Fig 5B).

**Fig 6:**
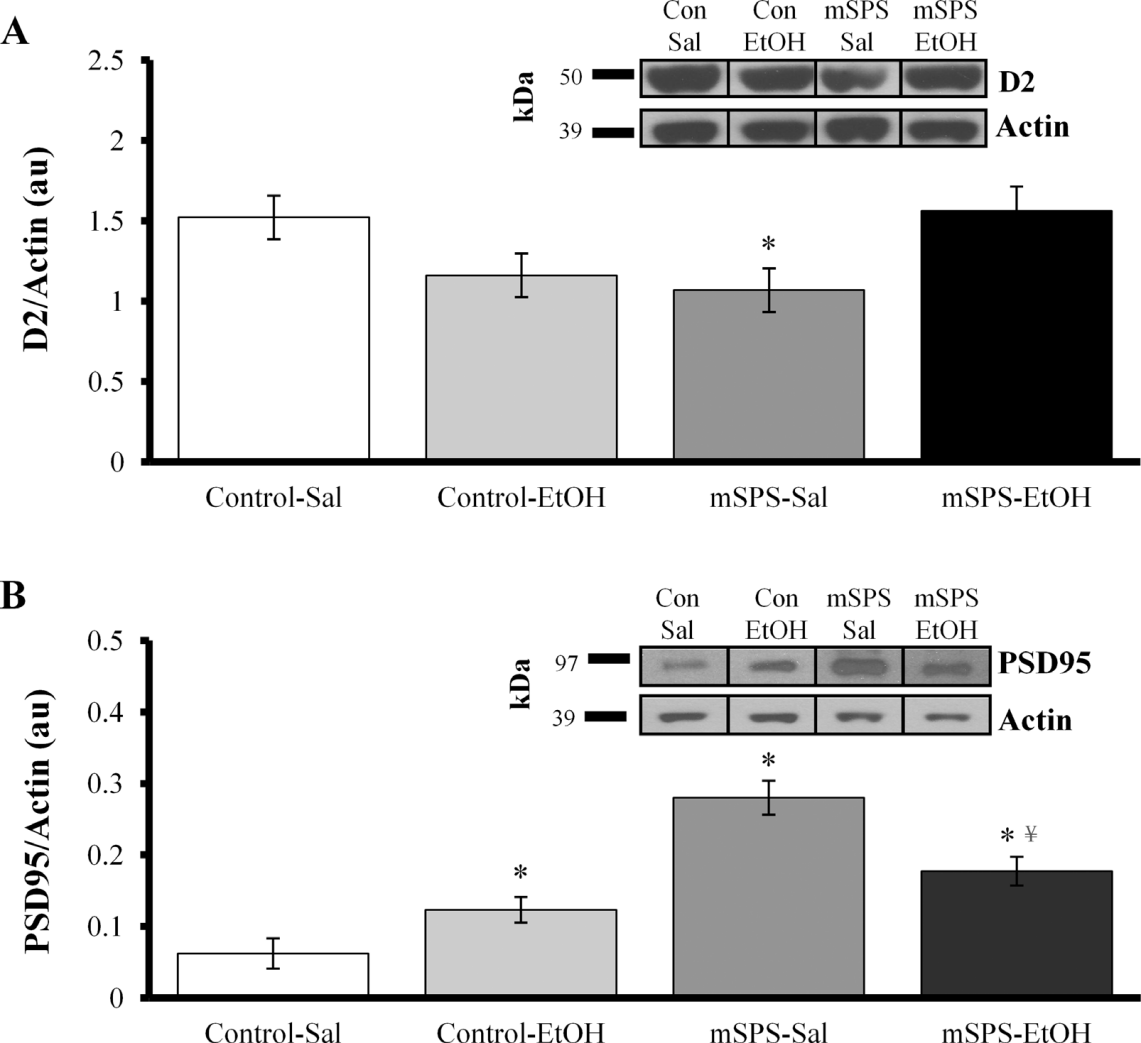
Interactions between mSPS and EtOH on D2-receptor and PSD95 protein levels. (A) Striatal D2 receptors were significantly decreased in mSPS-Sal mice compared to both Control-Saline and mSPS-EtOH mice (*p<0.05). Representative samples inserted in upper right corner (kDa, kilodaltons). (B) PSD95 was significantly increased in Control-EtOH, mSPS-Sal, and mSPS-EtOH mice compared to Control-Sal mice (*p<0.05). PSD95 was also significantly decreased in mSPS-EtOH mice compared to mSPS-Sal mice (^¥^*p*<0.05).

On a separate blot, a total of 19 samples were utilized for analysis of D2 receptors (1 mSPS-EtOH sample had been removed from the original 20 samples due to insufficient protein collection). Final groups included, Control-Sal (*n* = 5), Control-EtOH (*n* = 5), mSPS-Sal (*n* = 5), and mSPS-EtOH (*n* = 4). Analysis of D2 receptors with a two-way ANOVA revealed a significant mSPS*EtOH interaction, *F*(3, 15) = 9.381, *p* = 0.008 (Fig 6A). Pair-wise comparisons indicated that D2 receptors were significantly decreased in mSPS-Sal mice compared to Control-Sal, *p* = 0.032, and mSPS-EtOH, *p* = 0.028, mice. Control-EtOH mice did not significantly differ from Control-Sal, *p* = 0.080, or mSPS-EtOH, *p* = 0.066, mice.

A total of 22 samples were utilized for PSD95 assays on the same blot as the CB1 assays. Final groups included, Control-Sal (*n* = 5), Control-EtOH (*n* = 7), mSPS-Sal (*n* = 4), and mSPS-EtOH (*n* = 6). Analysis of PSD95 using a two-way ANOVA indicated a significant mSPS*EtOH interaction, *F*(3,17) = 15.390, *p* = 0.001 (Fig 6B). Pair-wise comparisons indicated that PSD95 was significantly increased in Control-EtOH mice compared to Control-Sal mice, *p* = 0.045. Additionally, PSD95 was significantly increased in mSPS-Sal mice compared to Control-Sal, *p*<0.001, and mSPS-EtOH, *p* = 0.004, mice.

## Discussion

Overall, the present study demonstrates evidence of impaired striatal neuroplasticity in mSPS mice, as reflected by a disruption in EtOH-induced locomotor sensitization. The impaired behavioral response observed in mice exposed to both mSPS and EtOH was associated with a decrease in striatal CB1 receptors. Repeated EtOH exposure increased striatal PSD95 in accordance with previous reports [68, 73, 74]. Additionally, increases in striatal PSD95 and decreases in striatal D2 receptor proteins induced by mSPS were reversed in mice exposed to mSPS and EtOH.

Although the inability of mSPS mice to sensitize to EtOH suggests impaired striatal neuroplasticity, most studies of psychostimulant sensitization imply heightened sensitization is predictive of addictive behaviors [75–77]. However, several studies show that EtOH sensitization can also be reduced in rodents with increased EtOH preference or voluntary intake [78–81]. Specifically, C57BJL/6 mice, the mice used in this study, are typically more resistant to EtOH sensitization compared to DBA/2J mice [80], despite having increased EtOH preference (for review [82]). The reduced sensitization of mSPS mice may reflect an already maximized stress response. EtOH sensitization is modified by corticosterone and corticotrophin-releasing factor-1 receptors- both of which are important mechanisms of the stress response [83, 84]. SPS-exposed rats display elevated glucorticoid receptor mRNA expression in several regions of the brain and elevated corticotrophin releasing factor in the hypothalamus, and thus far our results indicate that glucorticoid receptor mRNA is upregulated in the hippocampus of mSPS-exposed mice [28, 85–87]. While mild stressors or injections of corticosterone enhance ethanol sensitivity [84], perhaps the severity of the mSPS combined with the stress of EtOH exposure has induced a ceiling effect, where EtOH-induced behavioral sensitization can no longer be detected. Correspondingly, what appears to be partial attenuation of increases in PSD95 in mSPS-EtOH exposed mice, may indicate that PSD95 can no longer compensate for the reduced glutamatergic tone, similar to that observed in chronic stress models of depression [88, 89].

This study indicates an interaction between mSPS and EtOH exposure, but the complexity of signaling within the striatum offers numerous interpretations of the data presented. First, CB1 receptors are localized to both glutamatergic and GABAergic synapses [90] suggesting that either or both cell populations may contribute to our observations. For instance, reductions of CB1 receptor expression on glutamatergic synapses would be expected to increase glutamatergic signaling, and therefore increase the activation of GABAergic MSNs. Alternatively, decreases in GABAergic CB1 receptor expression would lead to an increase in GABA release to MSNs, decreasing their activation. Second, CB1 receptors are thought to compensate for changes in endocannabinoid release [25], with a wide variety of mechanisms contributing to their release (for review see [50]). Finally, traumatic stress and chronic EtOH differentially impact the endocannabinoid system. In rodent studies, chronic EtOH exposure increases endocannabinoid release [37] while decreasing endocannabinoid signaling [39], and clinical studies show a decrease in CB1-binding in many brain regions, including the striatum of alcoholics [91]. Alternatively, stress rodent models suggest an initial increase in endocannabinoids with acute stress followed by decreases in endocannabinoids after chronic stress [17]. Further dissection of the effects of EtOH and traumatic stress on independent systems is needed to completely elucidate the mechanisms of this interaction.

Changes in PSD95 after both traumatic stress and EtOH exposure suggest the involvement of glutamatergic dysregulation. In response to decreases in glutamatergic signaling, PSD95 increases the clustering of NMDA receptors and the insertion and retention of α-amino-3-hydroxy-5-methyl-4-isoxazolepropionic acid (AMPA) receptors [64]. Elevated PSD95 with repeated EtOH corresponds to reported findings that PSD95 compensates for the ability of EtOH to antagonize NMDA receptors [73]. The increased PSD95 in Control-mSPS mice shown here is supportive of decreased glutamatergic tone in SPS rats reported in a previous study from our group [92]. Endocannabinoids are also known to indirectly alter NMDA receptors through their regulation of pre-synaptic glutamatergic neurons [93, 94], but additional studies are needed to determine if the decrease in CB1 occurs in response to decreased glutamatergic signaling or if it is related to another mechanism involved in endocannabinoid regulation, such as, changes in activation of adenylyl cyclases, cholinergic input, or dopaminergic tone [50].

Decreases in striatal D2 receptors after mSPS-exposure in mSPS-Sal mice were alleviated similarly to PSD95 in mSPS-EtOH mice. The decrease in D2 receptor protein after mSPS exposure corresponds to reported findings in rats exposed to SPS [61] and chronic stress [95]. Similarly, clinical studies on those with PTSD suggest genetic involvement of genes related to D2 receptors [96] and demonstrate elevated striatal dopamine-transporter protein [97], implying corresponding decreases in D2 receptors. D2 receptors were not significantly reduced in the assessed Control-EtOH mice. While D2 receptors are often decreased in those with alcoholism, some studies do not detect decreases in striatal D2 receptors ([60, 98] for review [99]). The alleviation of SPS-induced D2 receptor loss in EtOH exposed mice was not expected, and continued study is needed to elucidate more fully the mechanisms involved. Others report increased striatal D2 receptors in CB1-knockout mice [100], suggesting that D2 receptors are compensating for the dramatic loss of CB1 receptors. Interestingly, a subpopulation of alcoholics that is at particularly high risk of relapse displays an attenuation of decreased D2 compared to alcoholics who are able to recover with rehabilitation [59]. Determining if this high-risk population of alcoholics also has a greater history of traumatic stress would be an important future direction of study.

Overall, the present study suggests that severe, multimodal stress exposure impairs the striatal neuroplasticity that develops with prolonged EtOH treatment and that altered CB1 receptor expression is associated with this change. Furthermore, these findings point to CB1 receptors as a potential therapeutic target for the treatment of alcoholism in those affected with PTSD.

## Supporting Information

S1 Table Locomotor activity data. (DOCX)

S2 Table Immunoblot data. (DOCX)

## References

[1] Blanco C, Xu Y, Brady K, Perez-Fuentes G, Okuda M, Wang S (2013): Comorbidity of posttraumatic stress disorder with alcohol dependence among US adults: results from National Epidemiological Survey on Alcohol and Related Conditions. Drug Alcohol Depend. 132:630–638. 10.1016/j.drugalcdep.2013.04.016 23702490 PMC3770804

[2] Marshall BD, Prescott MR, Liberzon I, Tamburrino MB, Calabrese JR, Galea S (2012): Coincident posttraumatic stress disorder and depression predict alcohol abuse during and after deployment among Army National Guard soldiers. Drug Alcohol Depend. 124:193–199. 10.1016/j.drugalcdep.2011.12.027 22342428

[3] Bruijnzeel AW, Small E, Pasek TM, Yamada H (2010): Corticotropin-releasing factor mediates the dysphoria-like state associated with alcohol withdrawal in rats. Behav Brain Res. 210:288–291. 10.1016/j.bbr.2010.02.043 20193713 PMC3319063

[4] Bruijnzeel AW, Zislis G, Wilson C, Gold MS (2007): Antagonism of CRF receptors prevents the deficit in brain reward function associated with precipitated nicotine withdrawal in rats. Neuropsychopharmacology. 32:955–963. 10.1038/sj.npp.1301192 16943772

[5] Koob GF, Buck CL, Cohen A, Edwards S, Park PE, Schlosburg JE, et al (2014): Addiction as a stress surfeit disorder. Neuropharmacology. 76 Pt B:370–382. 10.1016/j.neuropharm.2013.05.024 23747571 PMC3830720

[6] Richardson HN, Zhao Y, Fekete EM, Funk CK, Wirsching P, Janda KD, et al (2008): MPZP: a novel small molecule corticotropin-releasing factor type 1 receptor (CRF1) antagonist. Pharmacol Biochem Behav. 88:497–510. 10.1016/j.pbb.2007.10.008 18031798 PMC3319109

[7] Breese GR, Sinha R, Heilig M (2011): Chronic alcohol neuroadaptation and stress contribute to susceptibility for alcohol craving and relapse. Pharmacol Ther. 129:149–171. 10.1016/j.pharmthera.2010.09.007 20951730 PMC3026093

[8] Chung YA, Kim SH, Chung SK, Chae JH, Yang DW, Sohn HS, et al (2006): Alterations in cerebral perfusion in posttraumatic stress disorder patients without re-exposure to accident-related stimuli. Clin Neurophysiol. 117:637–642. 10.1016/j.clinph.2005.10.020 16426890

[9] Elman I, Lowen S, Frederick BB, Chi W, Becerra L, Pitman RK (2009): Functional neuroimaging of reward circuitry responsivity to monetary gains and losses in posttraumatic stress disorder. Biol Psychiatry. 66:1083–1090. 10.1016/j.biopsych.2009.06.006 19640506 PMC9446383

[10] Falconer E, Bryant R, Felmingham KL, Kemp AH, Gordon E, Peduto A, et al (2008): The neural networks of inhibitory control in posttraumatic stress disorder. J Psychiatry Neurosci. 33:413–422. 18787658 PMC2527717

[11] Grusser SM, Wrase J, Klein S, Hermann D, Smolka MN, Ruf M, et al (2004): Cue-induced activation of the striatum and medial prefrontal cortex is associated with subsequent relapse in abstinent alcoholics. Psychopharmacology (Berl). 175:296–302. 10.1007/s00213-004-1828-4 15127179

[12] Sachinvala N, Kling A, Suffin S, Lake R, Cohen M (2000): Increased regional cerebral perfusion by 99mTc hexamethyl propylene amine oxime single photon emission computed tomography in post-traumatic stress disorder. Mil Med. 165:473–479. 10870367

[13] Gerdeman GL, Ronesi J, Lovinger DM (2002): Postsynaptic endocannabinoid release is critical to long-term depression in the striatum. Nature Neuroscience. 5:446–451. 10.1038/nn832 11976704

[14] Adermark L, Talani G, Lovinger DM (2009): Endocannabinoid-dependent plasticity at GABAergic and glutamatergic synapses in the striatum is regulated by synaptic activity. Eur J Neurosci. 29:32–41. 10.1111/j.1460-9568.2008.06551.x 19120438 PMC2661034

[15] Rabinak CA, Angstadt M, Lyons M, Mori S, Milad MR, Liberzon I, et al (2014): Cannabinoid modulation of prefrontal-limbic activation during fear extinction learning and recall in humans. Neurobiol Learn Mem. 113:125–134. 10.1016/j.nlm.2013.09.009 24055595 PMC3960373

[16] Varvel SA, Wise LE, Niyuhire F, Cravatt BF, Lichtman AH (2007): Inhibition of fatty-acid amide hydrolase accelerates acquisition and extinction rates in a spatial memory task. Neuropsychopharmacology. 32:1032–1041. 10.1038/sj.npp.1301224 17047668

[17] Akirav I (2013): Cannabinoids and glucocorticoids modulate emotional memory after stress. Neurosci Biobehav Rev. 37:2554–2563. 10.1016/j.neubiorev.2013.08.002 23954749

[18] Barna I, Zelena D, Arszovszki AC, Ledent C (2004): The role of endogenous cannabinoids in the hypothalamo-pituitary-adrenal axis regulation: in vivo and in vitro studies in CB1 receptor knockout mice. Life Sci. 75:2959–2970. 10.1016/j.lfs.2004.06.006 15454346

[19] Bowles NP, Hill MN, Bhagat SM, Karatsoreos IN, Hillard CJ, McEwen BS (2012): Chronic, noninvasive glucocorticoid administration suppresses limbic endocannabinoid signaling in mice. Neuroscience. 204:83–89. 10.1016/j.neuroscience.2011.08.048 21939741 PMC3697830

[20] Di S, Malcher-Lopes R, Halmos KC, Tasker JG (2003): Nongenomic glucocorticoid inhibition via endocannabinoid release in the hypothalamus: a fast feedback mechanism. J Neurosci. 23:4850–4857. 12832507 10.1523/JNEUROSCI.23-12-04850.2003 PMC6741208

[21] Gunduz-Cinar O, MacPherson KP, Cinar R, Gamble-George J, Sugden K, Williams B, et al (2013): Convergent translational evidence of a role for anandamide in amygdala-mediated fear extinction, threat processing and stress-reactivity. Mol Psychiatry. 18:813–823. 10.1038/mp.2012.72 22688188 PMC3549323

[22] Agosti V, Nunes E, Levin F (2002): Rates of psychiatric comorbidity among U.S. residents with lifetime cannabis dependence. Am J Drug Alcohol Abuse. 28:643–652. 12492261 10.1081/ada-120015873

[23] Cougle JR, Bonn-Miller MO, Vujanovic AA, Zvolensky MJ, Hawkins KA (2011): Posttraumatic stress disorder and cannabis use in a nationally representative sample. Psychol Addict Behav. 25:554–558. 10.1037/a0023076 21480682

[24] Potter CM, Vujanovic AA, Marshall-Berenz EC, Bernstein A, Bonn-Miller MO (2011): Posttraumatic stress and marijuana use coping motives: the mediating role of distress tolerance. J Anxiety Disord. 25:437–443. 10.1016/j.janxdis.2010.11.007 21146357 PMC3101637

[25] Neumeister A, Normandin MD, Pietrzak RH, Piomelli D, Zheng MQ, Gujarro-Anton A, et al (2013): Elevated brain cannabinoid CB1 receptor availability in post-traumatic stress disorder: a positron emission tomography study. Mol Psychiatry. 18:1034–1040. 10.1038/mp.2013.61 23670490 PMC3752332

[26] Jetly R, Heber A, Fraser G, Boisvert D (2015): The efficacy of nabilone, a synthetic cannabinoid, in the treatment of PTSD-associated nightmares: A preliminary randomized, double-blind, placebo-controlled cross-over design study. Psychoneuroendocrinology. 51C:585–588. 10.1016/j.psyneuen.2014.11.002 25467221

[27] Hauer D, Schelling G, Gola H, Campolongo P, Morath J, Roozendaal B, et al (2013): Plasma concentrations of endocannabinoids and related primary fatty acid amides in patients with post-traumatic stress disorder. PLoS One. 8:e62741 10.1371/journal.pone.0062741 23667516 PMC3647054

[28] Eagle AL, Mulo K., Kohler R. J., Conti A. C., and Perrine S. A. (2013): Single prolonged stress: Validity of translating PTSD model into mice San Diego, CA, U.S.A.: Society for Neuroscience. url

[29] Liberzon I, Krstov M, Young EA (1997): Stress-restress: effects on ACTH and fast feedback. Psychoneuroendocrinology. 22:443–453. 9364622 10.1016/s0306-4530(97)00044-9

[30] Ganon-Elazar E, Akirav I (2012): Cannabinoids prevent the development of behavioral and endocrine alterations in a rat model of intense stress. Neuropsychopharmacology. 37:456–466. 10.1038/npp.2011.204 21918506 PMC3242307

[31] Ganon-Elazar E, Akirav I (2013): Cannabinoids and traumatic stress modulation of contextual fear extinction and GR expression in the amygdala-hippocampal-prefrontal circuit. Psychoneuroendocrinology. 38:1675–1687. 10.1016/j.psyneuen.2013.01.014 23433741

[32] Roberto M, Cruz M, Bajo M, Siggins GR, Parsons LH, Schweitzer P (2010): The endocannabinoid system tonically regulates inhibitory transmission and depresses the effect of ethanol in central amygdala. Neuropsychopharmacology. 35:1962–1972. 10.1038/npp.2010.70 20463657 PMC2904853

[33] Basavarajappa BS, Ninan I, Arancio O (2008): Acute ethanol suppresses glutamatergic neurotransmission through endocannabinoids in hippocampal neurons. J Neurochem. 107:1001–1013. 10.1111/j.1471-4159.2008.05685.x 18796007 PMC2585363

[34] Lovinger DM, Roberto M (2013): Synaptic effects induced by alcohol. Curr Top Behav Neurosci. 13:31–86. 10.1007/7854_2011_143 21786203 PMC4791588

[35] Yin HH, Park BS, Adermark L, Lovinger DM (2007): Ethanol reverses the direction of long-term synaptic plasticity in the dorsomedial striatum. Eur J Neurosci. 25:3226–3232. 10.1111/j.1460-9568.2007.05606.x 17552991

[36] Vinod KY, Kassir SA, Hungund BL, Cooper TB, Mann JJ, Arango V (2010): Selective alterations of the CB1 receptors and the fatty acid amide hydrolase in the ventral striatum of alcoholics and suicides. J Psychiatr Res. 44:591–597. 10.1016/j.jpsychires.2009.11.013 20015515 PMC2878847

[37] DePoy L, Daut R, Brigman JL, MacPherson K, Crowley N, Gunduz-Cinar O, et al (2013): Chronic alcohol produces neuroadaptations to prime dorsal striatal learning. Proc Natl Acad Sci U S A. 110:14783–14788. 10.1073/pnas.1308198110 23959891 PMC3767559

[38] Vinod KY, Yalamanchili R, Xie S, Cooper TB, Hungund BL (2006): Effect of chronic ethanol exposure and its withdrawal on the endocannabinoid system. Neurochem Int. 49:619–625. 10.1016/j.neuint.2006.05.002 16822589

[39] Adermark L, Jonsson S, Ericson M, Soderpalm B (2011): Intermittent ethanol consumption depresses endocannabinoid-signaling in the dorsolateral striatum of rat. Neuropharmacology. 61:1160–1165. 10.1016/j.neuropharm.2011.01.014 21251919

[40] Lallemand F, Soubrie PH, De Witte PH (2001): Effects of CB1 cannabinoid receptor blockade on ethanol preference after chronic ethanol administration. Alcohol Clin Exp Res. 25:1317–1323. 11584151

[41] Lessov CN, Phillips TJ (1998): Duration of sensitization to the locomotor stimulant effects of ethanol in mice. Psychopharmacology (Berl). 135:374–382. 9539262 10.1007/s002130050525

[42] Phillips TJ, Burkhart-Kasch S, Crabbe JC (1991): Locomotor activity response to chronic ethanol treatment in selectively bred FAST and SLOW mice. Alcohol Alcohol Suppl. 1:109–113. 1845525

[43] Koob GF (2008): Corticotropin-releasing factor, neuroplasticity (sensitization), and alcoholism. Proc Natl Acad Sci U S A. 105:8809–8810. 10.1073/pnas.0804354105 18583480 PMC2449322

[44] Souza-Formigoni ML, De Lucca EM, Hipolide DC, Enns SC, Oliveira MG, Nobrega JN (1999): Sensitization to ethanol's stimulant effect is associated with region-specific increases in brain D2 receptor binding. Psychopharmacology (Berl). 146:262–267. 10541725 10.1007/s002130051115

[45] Coelhoso CC, Engelke DS, Filev R, Silveira DX, Mello LE, Santos-Junior JG (2013): Temporal and behavioral variability in cannabinoid receptor expression in outbred mice submitted to ethanol-induced locomotor sensitization paradigm. Alcohol Clin Exp Res. 37:1516–1526. 10.1111/acer.12130 23647533

[46] Basavarajappa BS, Yalamanchili R, Cravatt BF, Cooper TB, Hungund BL (2006): Increased ethanol consumption and preference and decreased ethanol sensitivity in female FAAH knockout mice. Neuropharmacology. 50:834–844. 10.1016/j.neuropharm.2005.12.005 16448676

[47] Blednov YA, Cravatt BF, Boehm SL 2nd, Walker D, Harris RA (2007): Role of endocannabinoids in alcohol consumption and intoxication: studies of mice lacking fatty acid amide hydrolase. Neuropsychopharmacology. 32:1570–1582. 10.1038/sj.npp.1301274 17164820

[48] Naassila M, Pierrefiche O, Ledent C, Daoust M (2004): Decreased alcohol self-administration and increased alcohol sensitivity and withdrawal in CB1 receptor knockout mice. Neuropharmacology. 46:243–253. 14680762 10.1016/j.neuropharm.2003.09.002

[49] Racz I, Bilkei-Gorzo A, Toth ZE, Michel K, Palkovits M, Zimmer A (2003): A critical role for the cannabinoid CB1 receptors in alcohol dependence and stress-stimulated ethanol drinking. J Neurosci. 23:2453–2458. 12657705 10.1523/JNEUROSCI.23-06-02453.2003 PMC6742040

[50] Mathur BN, Lovinger DM (2012): Endocannabinoid-dopamine interactions in striatal synaptic plasticity. Front Pharmacol. 3:66 10.3389/fphar.2012.00066 22529814 PMC3329863

[51] Blume LC, Bass CE, Childers SR, Dalton GD, Roberts DC, Richardson JM, et al (2013): Striatal CB1 and D2 receptors regulate expression of each other, CRIP1A and delta opioid systems. J Neurochem. 124:808–820. 10.1111/jnc.12139 23286559 PMC3697910

[52] Andre VM, Cepeda C, Cummings DM, Jocoy EL, Fisher YE, William Yang X, et al (2010): Dopamine modulation of excitatory currents in the striatum is dictated by the expression of D1 or D2 receptors and modified by endocannabinoids. Eur J Neurosci. 31:14–28. 10.1111/j.1460-9568.2009.07047.x 20092552

[53] Giuffrida A, Parsons LH, Kerr TM, Rodriguez de Fonseca F, Navarro M, Piomelli D (1999): Dopamine activation of endogenous cannabinoid signaling in dorsal striatum. Nature Neuroscience. 2:358–363. 10.1038/7268 10204543

[54] Yin HH, Lovinger DM (2006): Frequency-specific and D2 receptor-mediated inhibition of glutamate release by retrograde endocannabinoid signaling. Proc Natl Acad Sci U S A. 103:8251–8256. 10.1073/pnas.0510797103 16698932 PMC1472459

[55] Cunningham CL, Howard MA, Gill SJ, Rubinstein M, Low MJ, Grandy DK (2000): Ethanol-conditioned place preference is reduced in dopamine D2 receptor-deficient mice. Pharmacol Biochem Behav. 67:693–699. 11166059 10.1016/s0091-3057(00)00414-7

[56] Corbit LH, Nie H, Janak PH (2014): Habitual responding for alcohol depends upon both AMPA and D2 receptor signaling in the dorsolateral striatum. Front Behav Neurosci. 8:301 10.3389/fnbeh.2014.00301 25228865 PMC4151333

[57] Thanos PK, Gopez V, Delis F, Michaelides M, Grandy DK, Wang GJ, et al (2011): Upregulation of cannabinoid type 1 receptors in dopamine D2 receptor knockout mice is reversed by chronic forced ethanol consumption. Alcohol Clin Exp Res. 35:19–27. 10.1111/j.1530-0277.2010.01318.x 20958329 PMC3004984

[58] Ng GY, George SR (1994): Dopamine receptor agonist reduces ethanol self-administration in the ethanol-preferring C57BL/6J inbred mouse. Eur J Pharmacol. 269:365–374. 7895775 10.1016/0922-4106(94)90044-2

[59] Guardia J, Catafau AM, Batlle F, Martin JC, Segura L, Gonzalvo B, et al (2000): Striatal dopaminergic D(2) receptor density measured by [(123)I]iodobenzamide SPECT in the prediction of treatment outcome of alcohol-dependent patients. Am J Psychiatry. 157:127–129. 10618027 10.1176/ajp.157.1.127

[60] Volkow ND, Wang GJ, Fowler JS, Logan J, Hitzemann R, Ding YS, et al (1996): Decreases in dopamine receptors but not in dopamine transporters in alcoholics. Alcohol Clin Exp Res. 20:1594–1598. 8986209 10.1111/j.1530-0277.1996.tb05936.x

[61] Enman NM, Arthur K, Ward SJ, Perrine SA, Unterwald EM (2015): Anhedonia, Reduced Cocaine Reward, and Dopamine Dysfunction in a Rat Model of Posttraumatic Stress Disorder. Biol Psychiatry. 10.1016/j.biopsych.2015.04.024 PMC4644715 26115790

[62] Skelton K, Ressler KJ, Norrholm SD, Jovanovic T, Bradley-Davino B (2012): PTSD and gene variants: new pathways and new thinking. Neuropharmacology. 62:628–637. 10.1016/j.neuropharm.2011.02.013 21356219 PMC3136568

[63] Young RM, Lawford BR, Noble EP, Kann B, Wilkie A, Ritchie T, et al (2002): Harmful drinking in military veterans with post-traumatic stress disorder: association with the D2 dopamine receptor A1 allele. Alcohol Alcohol. 37:451–456. 12217937 10.1093/alcalc/37.5.451

[64] Keith D, El-Husseini A (2008): Excitation Control: Balancing PSD-95 Function at the Synapse. Front Mol Neurosci. 1:4 10.3389/neuro.02.004.2008 18946537 PMC2526002

[65] Ron D, Messing RO (2013): Signaling pathways mediating alcohol effects. Curr Top Behav Neurosci. 13:87–126. 10.1007/7854_2011_161 21877259 PMC3684072

[66] Welch JM, Wang D, Feng G (2004): Differential mRNA expression and protein localization of the SAP90/PSD-95-associated proteins (SAPAPs) in the nervous system of the mouse. J Comp Neurol. 472:24–39. 10.1002/cne.20060 15024750

[67] Chen M, Wan Y, Ade K, Ting J, Feng G, Calakos N (2011): Sapap3 deletion anomalously activates short-term endocannabinoid-mediated synaptic plasticity. J Neurosci. 31:9563–9573. 10.1523/JNEUROSCI.1701-11.2011 21715621 PMC3367431

[68] Shibasaki M, Mizuno K, Kurokawa K, Ohkuma S (2011): Enhancement of histone acetylation in midbrain of mice with ethanol physical dependence and its withdrawal. Synapse. 65:1244–1250. 10.1002/syn.20947 21538550

[69] Fitzgerald PJ, Pinard CR, Camp MC, Feyder M, Sah A, Bergstrom HC, et al (2014): Durable fear memories require PSD-95. Mol Psychiatry. 10.1038/mp.2014.161 25824301

[70] Hawley DF, Morch K, Christie BR, Leasure JL (2012): Differential response of hippocampal subregions to stress and learning. PLoS One. 7:e53126 10.1371/journal.pone.0053126 23285257 PMC3532167

[71] Sebastian V, Estil JB, Chen D, Schrott LM, Serrano PA (2013): Acute physiological stress promotes clustering of synaptic markers and alters spine morphology in the hippocampus. PLoS One. 8:e79077 10.1371/journal.pone.0079077 24205365 PMC3812005

[72] Conti AC, Maas JW Jr., Muglia LM, Dave BA, Vogt SK, Tran TT, et al (2007): Distinct regional and subcellular localization of adenylyl cyclases type 1 and 8 in mouse brain. Neuroscience. 146:713–729. 10.1016/j.neuroscience.2007.01.045 17335981 PMC1939925

[73] Camp MC, Feyder M, Ihne J, Palachick B, Hurd B, Karlsson RM, et al (2011): A novel role for PSD-95 in mediating ethanol intoxication, drinking and place preference. Addict Biol. 16:428–439. 10.1111/j.1369-1600.2010.00282.x 21309945 PMC3150485

[74] Suvarna N, Borgland SL, Wang J, Phamluong K, Auberson YP, Bonci A, et al (2005): Ethanol alters trafficking and functional N-methyl-D-aspartate receptor NR2 subunit ratio via H-Ras. J Biol Chem. 280:31450–31459. 10.1074/jbc.M504120200 16009711

[75] Belujon P, Grace AA (2011): Hippocampus, amygdala, and stress: interacting systems that affect susceptibility to addiction. Ann N Y Acad Sci. 1216:114–121. 10.1111/j.1749-6632.2010.05896.x 21272015 PMC3141575

[76] Prasad BM, Sorg BA, Ulibarri C, Kalivas PW (1995): Sensitization to stress and psychostimulants. Involvement of dopamine transmission versus the HPA axis. Ann N Y Acad Sci. 771:617–625. 8597435 10.1111/j.1749-6632.1995.tb44714.x

[77] Robinson TE, Becker JB (1986): Enduring changes in brain and behavior produced by chronic amphetamine administration: a review and evaluation of animal models of amphetamine psychosis. Brain Res. 396:157–198. 3527341 10.1016/s0006-8993(86)80193-7

[78] Cunningham CL, Niehus DR, Malott DH, Prather LK (1992): Genetic differences in the rewarding and activating effects of morphine and ethanol. Psychopharmacology (Berl). 107:385–393. 1352057 10.1007/BF02245166

[79] Phillips TJ, Crabbe JC, Metten P, Belknap JK (1994): Localization of genes affecting alcohol drinking in mice. Alcohol Clin Exp Res. 18:931–941. 7978106 10.1111/j.1530-0277.1994.tb00062.x

[80] Phillips TJ, Dickinson S, Burkhart-Kasch S (1994): Behavioral sensitization to drug stimulant effects in C57BL/6J and DBA/2J inbred mice. Behav Neurosci. 108:789–803. 7986372 10.1037//0735-7044.108.4.789

[81] Phillips TJ, Huson M, Gwiazdon C, Burkhart-Kasch S, Shen EH (1995): Effects of acute and repeated ethanol exposures on the locomotor activity of BXD recombinant inbred mice. Alcohol Clin Exp Res. 19:269–278. 7625557 10.1111/j.1530-0277.1995.tb01502.x

[82] Crawley JN, Belknap JK, Collins A, Crabbe JC, Frankel W, Henderson N, et al (1997): Behavioral phenotypes of inbred mouse strains: implications and recommendations for molecular studies. Psychopharmacology (Berl). 132:107–124. 9266608 10.1007/s002130050327

[83] Pastor R, McKinnon CS, Scibelli AC, Burkhart-Kasch S, Reed C, Ryabinin AE, et al (2008): Corticotropin-releasing factor-1 receptor involvement in behavioral neuroadaptation to ethanol: a urocortin1-independent mechanism. Proc Natl Acad Sci U S A. 105:9070–9075. 10.1073/pnas.0710181105 18591672 PMC2449366

[84] Pastor R, Reed C, Meyer PJ, McKinnon C, Ryabinin AE, Phillips TJ (2012): Role of corticotropin-releasing factor and corticosterone in behavioral sensitization to ethanol. J Pharmacol Exp Ther. 341:455–463. 10.1124/jpet.111.190595 22333290 PMC3336812

[85] Knox D, Nault T, Henderson C, Liberzon I (2012): Glucocorticoid receptors and extinction retention deficits in the single prolonged stress model. Neuroscience. 223:163–173. 10.1016/j.neuroscience.2012.07.047 22863672

[86] Liberzon I, Lopez JF, Flagel SB, Vazquez DM, Young EA (1999): Differential regulation of hippocampal glucocorticoid receptors mRNA and fast feedback: relevance to post-traumatic stress disorder. J Neuroendocrinol. 11:11–17. 9918224 10.1046/j.1365-2826.1999.00288.x

[87] Wang HT, Han F, Shi YX (2009): Activity of the 5-HT1A receptor is involved in the alteration of glucocorticoid receptor in hippocampus and corticotropin-releasing factor in hypothalamus in SPS rats. Int J Mol Med. 24:227–231. 19578795 10.3892/ijmm_00000225

[88] Cohen JW, Louneva N, Han LY, Hodes GE, Wilson RS, Bennett DA, et al (2011): Chronic corticosterone exposure alters postsynaptic protein levels of PSD-95, NR1, and synaptopodin in the mouse brain. Synapse. 65:763–770. 10.1002/syn.20900 21190219 PMC3401554

[89] Luo J, Zhang L, Ning N, Jiang H, Yu SY (2013): Neotrofin reverses the effects of chronic unpredictable mild stress on behavior via regulating BDNF, PSD-95 and synaptophysin expression in rat. Behav Brain Res. 253:48–53. 10.1016/j.bbr.2013.07.014 23850356

[90] Uchigashima M, Narushima M, Fukaya M, Katona I, Kano M, Watanabe M (2007): Subcellular arrangement of molecules for 2-arachidonoyl-glycerol-mediated retrograde signaling and its physiological contribution to synaptic modulation in the striatum. J Neurosci. 27:3663–3676. 10.1523/JNEUROSCI.0448-07.2007 17409230 PMC6672418

[91] Hirvonen J, Zanotti-Fregonara P, Umhau JC, George DT, Rallis-Frutos D, Lyoo CH, et al (2013): Reduced cannabinoid CB1 receptor binding in alcohol dependence measured with positron emission tomography. Mol Psychiatry. 18:916–921. 10.1038/mp.2012.100 22776901 PMC3594469

[92] Knox D, Perrine SA, George SA, Galloway MP, Liberzon I (2010): Single prolonged stress decreases glutamate, glutamine, and creatine concentrations in the rat medial prefrontal cortex. Neurosci Lett. 480:16–20. 10.1016/j.neulet.2010.05.052 20546834 PMC2902659

[93] Hampson RE, Miller F, Palchik G, Deadwyler SA (2011): Cannabinoid receptor activation modifies NMDA receptor mediated release of intracellular calcium: implications for endocannabinoid control of hippocampal neural plasticity. Neuropharmacology. 60:944–952. 10.1016/j.neuropharm.2011.01.039 21288475 PMC3059900

[94] Kauer JA, Malenka RC (2007): Synaptic plasticity and addiction. Nat Rev Neurosci. 8:844–858. 10.1038/nrn2234 17948030

[95] Yaroslavsky I, Tejani-Butt SM (2010): Voluntary alcohol consumption alters stress-induced changes in dopamine-2 receptor binding in Wistar-Kyoto rat brain. Pharmacol Biochem Behav. 94:471–476. 10.1016/j.pbb.2009.10.010 19896970 PMC2791177

[96] Comings DE, Muhleman D, Gysin R (1996): Dopamine D2 receptor (DRD2) gene and susceptibility to posttraumatic stress disorder: a study and replication. Biol Psychiatry. 40:368–372. 10.1016/0006-3223(95)00519-6 8874837

[97] Hoexter MQ, Fadel G, Felicio AC, Calzavara MB, Batista IR, Reis MA, et al (2012): Higher striatal dopamine transporter density in PTSD: an in vivo SPECT study with [(99m)Tc]TRODAT-1. Psychopharmacology (Berl). 224:337–345. 10.1007/s00213-012-2755-4 22700036

[98] Hietala J, West C, Syvalahti E, Nagren K, Lehikoinen P, Sonninen P, et al (1994): Striatal D2 dopamine receptor binding characteristics in vivo in patients with alcohol dependence. Psychopharmacology (Berl). 116:285–290. 7892418 10.1007/BF02245330

[99] Cosgrove KP (2010): Imaging receptor changes in human drug abusers. Curr Top Behav Neurosci. 3:199–217. 10.1007/7854_2009_24 21161754 PMC3760378

[100] Houchi H, Babovic D, Pierrefiche O, Ledent C, Daoust M, Naassila M (2005): CB1 receptor knockout mice display reduced ethanol-induced conditioned place preference and increased striatal dopamine D2 receptors. Neuropsychopharmacology. 30:339–349. 10.1038/sj.npp.1300568 15383833

