# Supplementary material for: Impaired Ethanol-Induced Sensitization and Decreased Cannabinoid Receptor-1 in a Model of Posttraumatic Stress Disorder
Source: PLoS One. 2016 May 17;11(5):e0155759. doi: 10.1371/journal.pone.0155759 (PMC4871361; doi:10.1371/journal.pone.0155759)
Supplement: S1 Table — (DOCX) [file pone.0155759.s001.docx]

| **Cohort** | **EtOH** | **mSPS** | **LMA (Total Beam Breaks)** | | | | **% Baseline LMA** | |
| --- | --- | --- | --- | --- | --- | --- | --- | --- |
|  |  |  | **Habituation** | **Day 1** | **Challenge** | | **Day 1** | **Challenge** |
| BEC-Test | EtOH | mSPS | 5848 | 1174 | 881 | | 142.82 | 107.18 |
| BEC-Test | Saline | mSPS | 6529 | 977 | 718 | | 243.03 | 178.61 |
| BEC-Test | Saline | Control | 5953 | 909 | 1415 | | 118.36 | 184.24 |
| BEC-Test | Saline | Control | 7715 | 1679 | 1427 | | 196.37 | 166.9 |
| BEC-Test | Saline | mSPS | 7863 | 1536 | 973 | | 179.23 | 113.54 |
| BEC-Test | Saline | mSPS | 4369 | 1220 | 1253 | | 884.06 | 907.97 |
| BEC-Test | EtOH | Control | 3822 | 786 | 1008 | | 845.16 | 1083.87 |
| BEC-Test | Saline | Control | 8289 | 846 | 1234 | | 103.05 | 150.3 |
| BEC-Test | EtOH | Control | 2667 | 748 | 1014 | | 699.07 | 947.66 |
| BEC-Test | EtOH | mSPS | 4896 | 612 | 915 | | 204.68 | 306.02 |
| BEC-Test | Saline | mSPS | 7286 | 1190 | 1252 | | 92.9 | 97.74 |
| BEC-Test | EtOH | mSPS | 10337 | 1010 | 983 | | 83.4 | 81.17 |
| BEC-Test | Saline | Control | 4504 | 969 | 1108 | | 434.53 | 496.86 |
| BEC-Test | Saline | Control | 7009 | 958 | 1420 | | 230.29 | 341.35 |
| BEC-Test | Saline | Control | 7640 | 1300 | 1193 | | 173.8 | 159.49 |
| BEC-Test | EtOH | Control | 4729 | 812 | 1590 | | 350 | 685.34 |
| BEC-Test | EtOH | Control | 5304 | 714 | 1049 | | 113.15 | 166.24 |
| BEC-Test | EtOH | Control | 3774 | 1152 | 2140 | | 702.44 | 1304.88 |
| BEC-Test | Saline | Control | 6180 | 1110 | 1023 | | 172.36 | 158.85 |
| BEC-Test | EtOH | mSPS | 6768 | 1811 | 1423 | | 194.52 | 152.85 |
| BEC-Test | EtOH | mSPS | 6937 | 1887 | 1383 | | 234.41 | 171.8 |
| BEC-Test | Saline | mSPS | 6567 | 1990 | 1375 | | 227.95 | 157.5 |
| BEC-Test | EtOH | mSPS | 5479 | 1349 | 997 | | 1284.76 | 949.52 |
| BEC-Test | EtOH | Control | 5883 | 1282 | 1357 | | 454.61 | 481.21 |
| BEC-Test | Saline | Control | 5543 | 868 | 828 | | 452.08 | 431.25 |
| BEC-Test | EtOH | Control | 4283 | 823 | 1054 | | 362.56 | 464.32 |
| BEC-Test | EtOH | Control | 4421 | 513 | 991 | | 1193.02 | 2304.65 |
| BEC-Test | Saline | mSPS | 8281 | 1711 | 1280 | | 318.03 | 237.92 |
| BEC-Test | EtOH | mSPS | 6907 | 2093 | 1622 | | 270.41 | 209.56 |
| BEC-Test | Saline | mSPS | 5994 | 695 | 828 | | 631.82 | 752.73 |
| BEC-Test | EtOH | mSPS | 6228 | 1764 | 1069 | | 459.38 | 278.39 |
| BEC-Test | Saline | mSPS | 10806 | 1627 | 2206 | | 133.03 | 180.38 |
| Immuno-Home | Saline | Control | 7933 | 654 | 799 | | 60.72 | 74.19 |
| Immuno-Home | EtOH | Control | 7150 | 793 | 1858 | | 143.92 | 337.21 |
| Immuno-Home | Saline | Control | 4587 | 737 | 750 | | 87.12 | 88.65 |
| Immuno-Home | EtOH | Control | 6370 | 1255 | 3027 | | 124.13 | 299.41 |
| Immuno-Home | Saline | mSPS | 2713 | 371 | 665 | | 421.59 | 755.68 |
| Immuno-Home | EtOH | mSPS | 5680 | 773 | 1426 | | 121.35 | 223.86 |
| Immuno-Home | Saline | mSPS | 6506 | 922 | 1187 | | 160.91 | 207.16 |
| **Cohort** | **EtOH** | **mSPS** | **LMA (Total Beam Breaks)** | | | | **% Baseline LMA** | |
|  |  |  | **Habituation** | **Day 1** | | **Challenge** | **Day 1** | **Challenge** |
| Immuno-Home | EtOH | mSPS | 10560 | 1111 | | 1611 | 124.13 | 180 |
| Immuno-Home | Saline | mSPS | 7667 | 619 | | 1880 | 79.05 | 240.1 |
| Immuno-Home | Saline | Control | 8190 | 1191 | | 754 | 169.66 | 107.41 |
| Immuno-Home | EtOH | Control | 2128 | 1248 | | 1051 | 630.3 | 530.81 |
| Immuno-Home | EtOH | Control | 8691 | 974 | | 2345 | 141.98 | 341.84 |
| Immuno-Home | Saline | Control | 7586 | 1333 | | 1737 | 208.28 | 271.41 |
| Immuno-Home | EtOH | mSPS | 3969 | 1157 | | 1733 | 273.52 | 409.69 |
| Immuno-Home | Saline | mSPS | 5325 | 686 | | 835 | 181.48 | 220.9 |
| Immuno-Home | EtOH | mSPS | 4716 | 1081 | | 1111 | 222.89 | 229.07 |
| Immuno-Home | Saline | mSPS | 9600 | 1539 | | 1013 | 125.94 | 82.9 |
| Immuno-Home | Saline | mSPS | 9775 | 1615 | | 1118 | 175.16 | 121.26 |
| Immuno-Home | EtOH | mSPS | 7987 | 1682 | | 1988 | 241.32 | 285.22 |
| Immuno-Home | EtOH | mSPS | 13296 | 1825 | | 882 | 112.03 | 54.14 |
| Immuno-Home | Saline | Control | 3113 | 815 | | 794 | 319.61 | 311.37 |
| Immuno-Home | EtOH | Control | 6556 | 1127 | | 2463 | 185.06 | 404.43 |
| Immuno-Home | Saline | Control | 4139 | 922 | | 719 | 922 | 719 |
| Immuno-Home | EtOH | Control | 4557 | 451 | | 1421 | 82.45 | 259.78 |
| Immuno-Home | Saline | mSPS | 7064 | 837 | | 1069 | 228.69 | 292.08 |
| Immuno-Home | EtOH | mSPS | 5393 | 1209 | | 1496 | 286.49 | 354.5 |
| Immuno-Home | Saline | mSPS | 7061 | 1562 | | 1161 | 548.07 | 407.37 |
| Immuno-Home | Saline | Control | 5640 | 995 | | 1060 | 214.9 | 228.94 |
| Immuno-Home | EtOH | Control | 6232 | 951 | | 3370 | 144.31 | 511.38 |
| Immuno-Home | Saline | Control | 5663 | 1159 | | 834 | 218.68 | 157.36 |
| Immuno-Home | Saline | Control | 4045 | 997 | | 978 | 1917.31 | 1880.77 |
| Immuno-Home | EtOH | mSPS | 4097 | 1384 | | 1366 | 499.64 | 493.14 |
| Immuno-Home | EtOH | mSPS | 10550 | 1145 | | 1637 | 105.05 | 150.18 |
| Immuno-Home | Saline | mSPS | 5627 | 1254 | | 910 | 245.88 | 178.43 |
| Immuno-Home | Saline | mSPS | 4427 | 834 | | 717 | 160.08 | 137.62 |
| Immuno-Home | EtOH | mSPS | 7227 | 1163 | | 1716 | 158.23 | 233.47 |
| Immuno-Home | EtOH | Control | 7443 | 1789 | | 2004 | 1569.3 | 1757.89 |
| Immuno-Home | Saline | Control | 7140 | 1808 | | 1048 | 439.9 | 254.99 |
| Immuno-Home | EtOH | Control | 6149 | 2167 | | 1313 | 268.53 | 162.7 |
| Immuno-Home | EtOH | Control | 6420 | 1008 | | 1326 | 238.86 | 314.22 |
