# Supplementary material for: Impaired Ethanol-Induced Sensitization and Decreased Cannabinoid Receptor-1 in a Model of Posttraumatic Stress Disorder
Source: PLoS One. 2016 May 17;11(5):e0155759. doi: 10.1371/journal.pone.0155759 (PMC4871361; doi:10.1371/journal.pone.0155759)
Supplement: S2 Table — (DOCX) [file pone.0155759.s002.docx]

| **Cohort** | **EtOH** | **mSPS** | **Immunoassays with Actin Control (Arbitrary Units)** | | |
| --- | --- | --- | --- | --- | --- |
|  |  |  | **CB1** | **PSD95** | **D2** |
| Immuno-Home | Saline | Control |  |  | 1.10 |
| Immuno-Home | EtOH | Control | 1.26 | 0.07 |  |
| Immuno-Home | Saline | Control | 1.02 | 0.07 |  |
| Immuno-Home | EtOH | Control | 1.43 | 0.13 |  |
| Immuno-Home | Saline | mSPS | 1.48 | 0.23 |  |
| Immuno-Home | EtOH | mSPS | 0.33 | 0.22 |  |
| Immuno-Home | Saline | mSPS |  |  | 1.18 |
| Immuno-Home | EtOH | mSPS | 0.82 | 0.21 |  |
| Immuno-Home | EtOH | mSPS | 0.31 | 0.20 |  |
| Immuno-Home | Saline | mSPS | 0.98 | 0.35 | 1.23 |
| Immuno-Home | Saline | Control | 1.34 | 0.15 |  |
| Immuno-Home | EtOH | Control | 1.06 | 0.13 |  |
| Immuno-Home | EtOH | Control | 1.04 | 0.13 |  |
| Immuno-Home | Saline | Control |  | 1.43 | 2.03 |
| Immuno-Home | EtOH | mSPS |  |  | 2.23 |
| Immuno-Home | Saline | mSPS |  |  | 1.05 |
| Immuno-Home | EtOH | mSPS |  | 0.20 | 1.16 |
| Immuno-Home | Saline | mSPS |  |  | 1.14 |
| Immuno-Home | Saline | mSPS |  |  |  |
| Immuno-Home | EtOH | mSPS | 0.25 | 0.10 |  |
| Immuno-Home | EtOH | mSPS | 0.37 | 0.13 |  |
| Immuno-Home | Saline | Control |  |  |  |
| Immuno-Home | EtOH | Control | 1.59 | 0.11 | 1.19 |
| Immuno-Home | Saline | Control | 1.68 | 0.002 | 1.71 |
| Immuno-Home | EtOH | Control | 0.69 | 0.10 |  |
| Immuno-Home | Saline | mSPS | 0.96 | 0.28 |  |
| Immuno-Home | EtOH | mSPS |  |  | 1.53 |
| Immuno-Home | Saline | mSPS | 1.09 | 0.26 |  |
| Immuno-Home | Saline | Control | 0.95 | 0.07 |  |
| Immuno-Home | EtOH | Control | 1.12 | 0.19 |  |
| Immuno-Home | EtOH | Control |  |  | 1.19 |
| Immuno-Home | Saline | Control |  |  | 1.24 |
| Immuno-Home | EtOH | mSPS |  |  | 1.33 |
| Immuno-Home | Saline | mSPS |  |  | 0.74 |
| Immuno-Home | EtOH | Control |  |  | 1.14 |
| Immuno-Home | Saline | Control |  |  | 1.52 |
| Immuno-Home | EtOH | Control |  |  | 1.09 |
| Immuno-Home | EtOH | Control |  |  | 1.19 |
